# Bed Bugs (Hemiptera, Cimicidae): Overview of Classification, Evolution and Dispersion

**DOI:** 10.3390/ijerph17124576

**Published:** 2020-06-25

**Authors:** Mohammad Akhoundi, Denis Sereno, Remy Durand, Asad Mirzaei, Christiane Bruel, Pascal Delaunay, Pierre Marty, Arezki Izri

**Affiliations:** 1 Parasitology-Mycology Department, Avicenne Hospital, AP-HP, Sorbonne Paris Nord University, 93000 Bobigny, France; remy.durand@aphp.fr (R.D.); arezki.izri@aphp.fr (A.I.); 2 Institut de Recherche pour le Développement, Montpellier University, MIVEGEC, 34032 Montpellier, France; denis.sereno@ird.fr (D.S.); delaunay.p@chu-nice.fr (P.D.); 3 Institut de Recherche pour le Développement, Montpellier University, InterTryp, 34032 Montpellier, France; 4 Parasitology Department, Paramedical School, Ilam University of Medical Sciences, 6931851147 Ilam, Iran; amirzaeii@yahoo.com; 5 Zoonotic Diseases Research Center, Ilam University of Medical Sciences, 6931851147 Ilam, Iran; 6 Agence Régionale de Santé (ARS) Île-de-France, 75935 Paris 19, France; christiane.bruel@ars.sante.fr; 7 Service Parasitologie-Mycologie, Centre Hospitalier Universitaire de Nice (CHU Nice), 06202 Nice, France; marty.p@chu-nice.fr; 8 Inserm U1065, Centre Méditerranéen de Médecine Moléculaire, C3 M, Université Côte d’Azur, 06204 Nice, France; 9 Unité des Virus Emergents (UVE: Aix Marseille Univ, IRD 190, INSERM 1207, IHU Méditerranée Infection), 13005 Marseille, France

**Keywords:** Ectoparasite, Fossil, Evolution, Dispersion, *C. lectularius*, *C. hemipterus*

## Abstract

The bed bugs (*Cimex lectularius* and *C. hemipterus*) have undergone a significant resurgence worldwide since the 1990s. A compilation of findings from a database, including 2650 scientific publications from seven major medical databases, allowed us to document main evolutionary events, from fossil evidence, dating from 11,000 years ago, until the present that has led to the current worldwide expansion of *Cimicid* species. We present the hypotheses on the possible dispersion pathways of bed bugs in light of the major historical and evolutionary events. A detailed classification of the Cimicidae family and finally, an illustrative map displaying the current distribution of known *Cimex* species in each geographical ecozone of Asia, Europe, Africa, the Americas, and Australia are presented.

## 1. Introduction

Bed bugs are obligate blood-sucking insects belonging to the Cimicidae family. They are ectoparasites with a long history of presence in human communities. They are a major concern to public health and currently one of the most common ectoparasites, affecting human life worldwide [1]. In recent decades, bed bug infestations in human habitats have drastically increased, leading to a rise in both nuisance and related disorders.

The presence of bed bug populations in endemic areas of Chagas disease has questioned the competence of these insects in the transmission of *Trypanosoma cruzi*, the etiological agent of the disease. As early as 1914, the experimental transmission of *T. cruzi* by *Cimex lectularius* has been ascertained in the laboratory by Blacklock [2] and reassessed more recently by Salazar et al. [3]. Nevertheless, no evidence supporting the role of *Cimex lectularius* in the transmission of *T. cruzi* in endemic areas is currently available. Currently, 40 infectious agents have been detected in bed bugs, including bacteria, fungi, viruses, parasites, filaroid nematodes, or protozoa [4]. The blood feeding habit of bed bugs causes a wide spectrum of dermatological manifestations, varying from erythematous macules or papules to bullous eruptions. Beside clinical issues, the presence of bed bugs in an infested location may occasionally leads to systemic and psychological disorders [5]. Finally, bed bugs are responsible for multiple economic problems that affect cultural and tourism industries (e.g., the economic impact of the resurgence was 100 million AUS dollars in Australia) [6].

The genus *Cimex* includes bug species with feeding preferences for a wide range of vertebrates from bats and birds to humans. The two cosmopolitan species, *C. lectularius* and *C. hemipterus*, feed almost exclusively on humans, and are responsible for significant infestation outbreaks. The development of DDT (dichlorodiphenyltrichloroethane) in 1939 has allowed the control of these ectoparasites, and consequently the reports on infestations sharply decreased in developed countries after World War II (WWII) [7]. Nevertheless, the eradication of these bugs in an infested location has been and is still a challenge, further puzzled by the emergence of insecticide resistant bug populations [8]. Indeed, since the 1990s, the formal reports of bed bugs resurgence in 135 countries from five continents pinpoint a serious problem for human wellness and health [7,8]. Therefore, information on the epidemiology, biology, physiology, and evolutive history of these bugs would allow modelisation of bed bug distribution for predictions regarding the future dispersal patterns and therefore will guide future interventions and control management strategies.

Here, we present an in-depth review of the literature published on bed bugs, with emphasis on the worldwide resurgence of the common bed bug, *C. lectularius* as well as the tropical bed bug, *C. hemipterus*. We talk over the Cimicids classification, and discuss the origin, evolution, and dispersion of these bugs.

## 2. Review of the Literature

**Figure 1:**
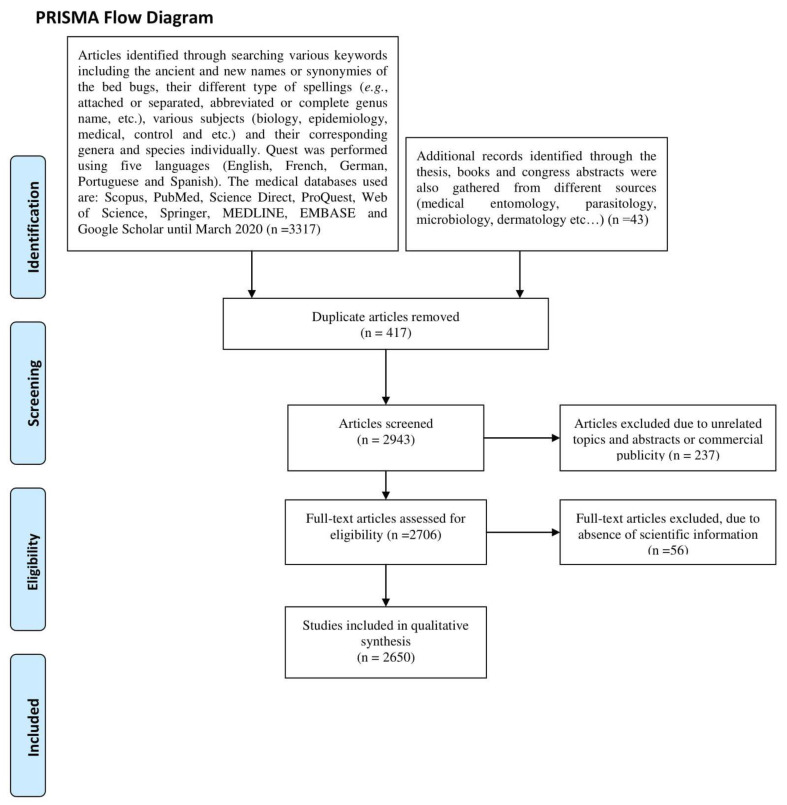
Schematic representation of literature quest strategy used in the present study.

During the past few years, an increasing number of publications, including research articles, books, and media, pinpoint a rise in incidence of bed bug infestations and highlight the consequences of such infestations on human health. To shed light on the past and present bed bug infestation as a worldwide medically important issue, a systematic review of the released literature, including research articles, books, and theses, was performed. The present survey relied on the PRISMA guideline (Preferred Reporting Items for Systematic Reviews and Meta-Analyses) [9]. Seven medical databases, including Scopus, PubMed, Science Direct, ProQuest, Web of Science, Springer, MEDLINE, EMBASE, and Google Scholar, were explored in articles published from 1890 to 2019. The searching strategy was performed, using keywords such as ancient and new names or synonymies of bed bugs, their spellings (e.g., attached or separated, abbreviated or complete genus name, etc.), their corresponding genera and species, and also including various scientific thematic issues of research (biology, epidemiology, medical, control, etc.). The quest was performed, using five languages (English, French, German, Portuguese and Spanish). The relevant articles that met the aforementioned criteria were selected. Duplicated articles, articles with unrelated topics and abstract or with commercial publicity were excluded. In addition, personal documents, from senior specialists were also included in the present study. Approximately 2650 articles published on bed bug related subjects were gathered (Figure 1, Table S1). 

**Figure 2:**
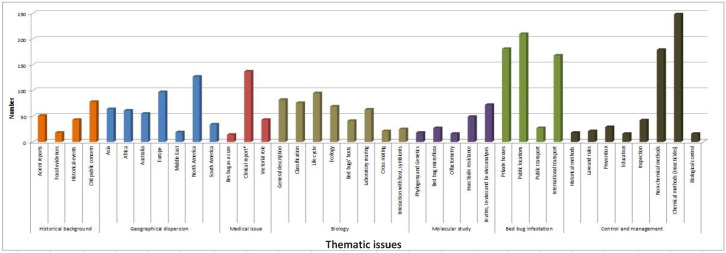
Thematic issues of the collected literature.

This literature was then categorized into seven thematic issues: historical background, geographical dispersion, medical issues, biology, molecular studies, bed bug infestations, control, and management. A synthetic view of the articles published within each defined theme is given in Figure 2.

## 3. Classification 

**Figure 3:**
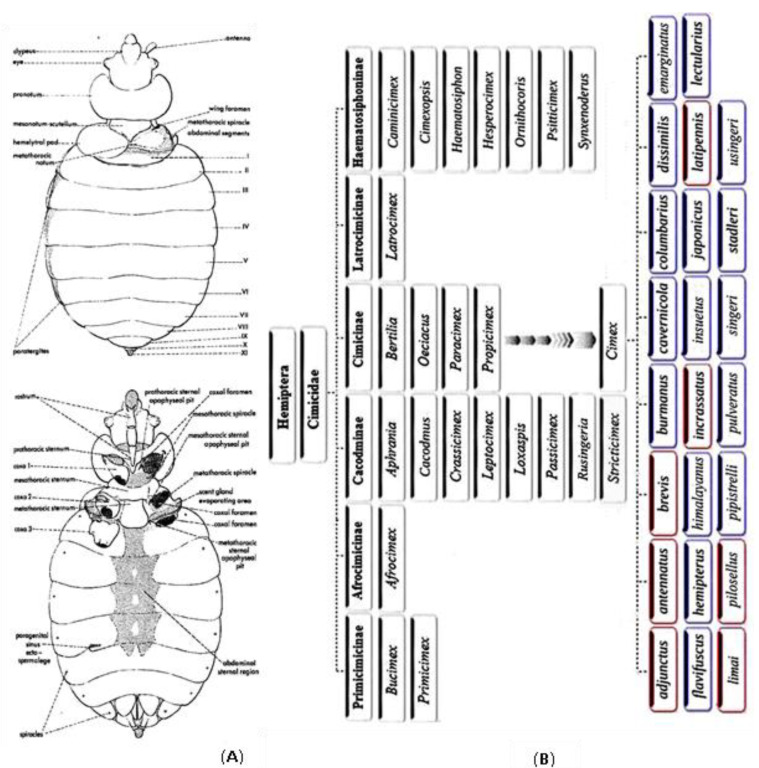
Panel (**A**) Morphological characteristics of bed bugs (*C. lectularius*), panel (**B**) Suggested updated classification of *Cimex* species. Species highlighted in blue and red have been likely originated/reported in the Old and New world, respectively.

Hematophagy has emerged in a minority of species within the broad class of *Insecta*, influencing the human health. Within the Hemiptera order and the Cimicomorpha infraorder (16 families), only the Cimicidae, Reduviidae (only Triatominae) and Polyctenidae families are of medical importance and hematophagous at all stages [10,11]. The Cimicidae family consists of six recognized subfamilies, 24 genera and 110 species. The biogeographical distribution shows continent-restricted distribution, with twelve genera which are exclusive to the new world, 9 restricted to the old world and 2 (*Cimex* and *Oeciocus*) both in the new and old worlds [12,13,14,15]. About two-thirds of the recognized species are associated with bats [16,17]. The phylogenetic relationship and the systematics of Cimicidae family were essentially based on morphological characters (in particular, genitalia), chromosome numbers, and their specialization to hosts [17,18]. The *Cimex* genus is traditionally divided into four groups of species, later supported by DNA analyses [17,19]: (i) *C. hemipterus* group, (ii) *C. lectularius* group, (iii) *C. pilosellus* group, and iv) *C. pipistrelli* group [17]. According to this classification, *C. lectularius* group includes *C. lectularius* and *C. columbarius* and *C. emarginatus* and *C. hemipterus* group consists of *C. hemipterus* and *C. insuetus* [19]. *Cimex* was shown to be paraphyletic with respect to the genera *Oeciacus* and *Paracimex*. Among *Cimex* species, *C. hemipterus* and *C. pipistrelli* group demonstrated to be closely related according to molecular analysis [19]. Within the Cimicidae family and Cimicinae subfamily, the genus *Cimex* includes 23 described species, 20 express trophic preference for bats, and 1 for birds. Three species routinely use humans as hosts but can feed occasionally on poultry or other domestic animals [17,20]. Of these, two belong to the Cimicinae subfamily, (*C. lectularius* and *C. hemipterus*) and one to the Cacodminae (*Leptocimex boueti*). Beside *C. lectularius* and *C. hemipterus*, *C. columbarius*, *C. pipistrelli*, *C. pilosellus*, *C. adjunctus* and *C. dissimilis* have been shown to feed occasionally on humans [21]. Within the Cimicinae subfamily, the currently described *Cimex* species include *C. adjunctus* (Barber 1939), *C. antennatus* (Usinger and Ueshima 1965), *C. brevis* (Usinger and Ueshima 1965), *C. burmanus* (Usinger 1966), *C. cavernicola* (Usinger 1966), *C. columbarius* (Jenyns 1839), *C. dissimilis* (Horváth 1910), *C. emarginatus* (Simov, Ivanova and Schunger, 2006), *C. flavifuscus* (Wendt 1939), *C. hemipterus* (Fabricius 1803), *C. himalayanus* (Bhat 1974), *C. incrassatus* (Usinger and Ueshima 1965), *C. insuetus* (Ueshima 1968), *C. japonicus* (Usinger 1966), *C. latipennis* (Usinger and Ueshima 1965), *C. lectularius* (Linnaeus 1758), *C. limai* (Pinto 1927), *C. pilosellus* (Horvath 1910), *C. pipistrelli* (Jenyns 1839), *C. pulveratus* (Hornok et al. 2018), *C. singeri* (China, 1938), *C. stadleri* (Horvath 1935) and *C. usingeri* (Bhat 1973) (Figure 3). The validity of these taxa remains a matter of debate mainly on the taxonomy at the sub-specific position [22,23] or on distinct valid species [17,24,25]. Here, we propose an inventory of *Cimex* species according to the trophic preferences:

### 3.1. Bugs that Feed on Humans: “Human Bugs”

*Cimex lectularius* is predominant in temperate regions and is by far the most common bug species reported in various countries [17]. Beside human dwelling infestation, it is observed in association with birds or bats in Europe [17,26], although it has never been recorded in association with bats in North America [17,27]. It is identified by diverse morphological characteristics e.g., broad lateral lobes of the pronotum, clefted and bristled paragenital sinus, width/length ratio of the pronotum more than two and head wider than the third antennal segment [17,28].

*Cimex hemipterus* (syn. *C. rotundatus*) referred to the tropical bed bug. It is frequently reported in several countries mostly in Southeast Asia, Africa, and Australia [7]. Width/length ratio of the pronotum is less than 2, lateral lobes of the pronotum are narrow and hind margins of hemelytral pads are broadly rounded on the inner halves [17]. 

### 3.2. Bugs that Feed on Birds: “Bird Bugs”

*Cimex columbarius* feed essentially on pigeons, probably after the domestication of pigeons (they have never been found in the cave nests of wild pigeons). *C. columbarius* is smaller than *C. lectularius* and has a relatively short third antennal segment [17,29]. The ratio of head width to the third antennal segment is 1.78 [17]. The taxonomic position of *C. columbarius* is still controversial. *C. columbarius* is considered as a subspecies of *C. lectularius* by Kassianoff [30] and Ueshima [31], whereas Titschack [32] and Usinger [17] promoted it to the species level. Because of morphological similarities with *C. lectularius*, it has been hypothesized that *C. lectularius* has evolved from *C. columbarius* [33]. Unlikely, based on other inverse theory, *C. columbarius* is reproductively isolated from *C. lectularius* [34]. 

### 3.3. Bugs that Feed on Bats: ″Bat Bugs″

They are split into two phylogenetic lineages [19]: the *Pilosellus* and the *Pipistrelli* groups.

(i) The *Pilosellus* group consists of *C. adjunctus*, *C. antennatus, C. brevis*, *C. incrassatus*, *C. latipennis*, and *C. pilosellus*, and inhabits the Nearctic region (North America) [19]. They are characterized by narrow lateral lobes of the pronotum, and rounded and bristled paragenital sinus.
(ii) The *Pipistrelli* group consists of 10 described species, including *C. burmanus*, *C. cavernicola*, *C. dissimilis*, *C. emarginatus*, *C. flavifuscus*, *C. japonicus*, *C. limai*, *C. pipistrelli*, *C. singeri*, and *C. stadleri*, which are dispersed throughout the Palaearctic region [17,35,36,37]. Species that belong to this group are characterized morphologically by a pronotum with narrow lateral lobes, and a naked paragenital sinus [19]. The members of the *Pipistrelli* group are morphologically differentiated among host bat species but molecular analyses highlight an absence of population structuring within these species [38,39]. However, crossing experiments performed in experimental settings suggest the existence of a reproductive barrier between the bat bugs populations belonging to the *Pipistrelli* group, and originating from the British Isles and those of the former Czechoslovakia. This observation has been recently challenged with molecular analysis [38]. These analyses support the existence of at least two separate lineages in Europe [17] with a different range of host preference [17,39,40]. *C. pipistrelli* group species have also been found in nests of birds [41]. In the Western Palaearctic region, *C. pipistrelli*, *C. dissimilis* and *C. stadleri* and one with an unclear taxonomic status (*C. pipistrelli* form *C. singeri* China, 1938) were described [17,25]. Stichel [42] and Péricart [43,44] considered *C. stadleri* as synonymous to *C. dissimilis*, while it is considered as a monotypic species by Wendt [22], Povolný [45], Lansbury [24], and Kerzhner [46].

#### 3.3.1. *Pilosellus* Group

*Cimex adjunctus* is mainly prevalent in the Eastern part of the Rocky Mountains in North America (entire Eastern USA, as well as Canada) [17,47]. In North America, various bat species roost temporarily together at night and this could potentially facilitate host switching [27,48]. It is also observed in tropical regions and in areas with a more temperate climate. This species feeds primarily on bats, although they do sometimes feed on humans, especially if its preferred blood source is no longer available. It has a pronotal width usually about 1.2 mm or more, long and thin bristles on the sides of the pronotum, the longest bristles of hind tibiae, being almost as long as the width of the tibia (0.90 mm) [17]. 

*Cimex antennatus* is a species found in North America. Its presence was reported for the first time in the fossils found in the Paisley caves in USA that date back to 5100 years ago [49]. Its presence in the caves implies that the climatic conditions at Paisley caves might be similar to those of the current habitats of *C. antennatus*. This species has a head relatively broad, pronotum less than 1.6 times, as wide as head and a haploid chromosome count of llA + XY [17]. Moreover, bristles on the sides of the pronotum subequal the width of the first antennal segment. It differentiated from *C. pilosellus* and *C. adjunctus* by a narrower pronotum and from *C. incrassatus* by a shorter bristle and a less incrassate hind femur [17]. 

*Cimex brevis* is present in the Midwest and Northeast of North America, as well as in Canada [50,51]. In this species, the longest bristles of the hind tibiae are longer than the width of tibia (1.25). It possesses a pronotum of 1.1 mm in width. It is closely related to *C. adjunctus* but can be differentiated by its smaller size and its long tibial bristles. *C. brevis* has been reported to transmit *Trypanosoma hedricki* in laboratory settings [52]. 

*Cimex incrassatus* is another North American *Cimex* species. It possesses the bristles longer than the width of the first antennal segment. It is somewhat similar to *C. antennatus* but distinguishable by its autosomes number. It has longer bristles, a stouter hind femora, and different antennal proportions. It is also similar to *C. pilosellus,* but can be differentiated by the wider hemelytral pads, a smaller size and a distinctive pattern of chromosome [17].

*Cimex latipennis* belongs to the group of bug species identified in the Paisley caves (USA) and dating back to 11 thousand years ago [49]. It is currently present both in the USA and Canada. The bristles in this species are usually less than 0.2 mm, thicker, and distinctly serrate. Furthermore, the longest bristles of hind tibiae in this species are shorter with hemelytral pads relatively short and broad, nearly twice as wide as they are long [17].

*Cimex limai* was described by Pinto in 1927 as the first ectoparasitic bug of bats in Brazil, but it can occasionally feed on humans [53]. It was the first representative of Cimicidae family, having ribaga and berlese organ localized in the center of the abdomen. It has a pronotum, ranging from 0.79 to 6 mm. Hemelytros presents a canonical appearance of the *Cimex* species, with a dorsal convex face and strongly coated with strong bristles, with 0.84 to 0.97 mm. External lateral bristles of hemelythros have from 0.095 to 0.097 mm length [53]. It was considered by Usinger [17] as a type of species of *Propicimex* genus. 

*Cimex pilosellus*, considered as a western bed bug, is almost present exclusively west of the Rocky Mountains in North America (western USA) and Canada [54]. This species feeds on several species of bats. The Hemelytral pads in this species are longer and narrower (width to length equals 1.6 to 1.7). It may be found both in urban and campestral settings, following their bat hosts and occasionally feeding on humans. On occasion, *C. pilosellus* can infest human residence where it can be easily confused with the common bed bug [55]. It has been reported to support the transmission of *T. cruzi* in laboratory settings [56]. 

#### 3.3.2. *Pipistrelli* Group

*Cimex burmanus* has been identified in Burma and is characterized by a short hind femora and a short third antennal segment [54]. In addition, hind femora are stout, 2.36 times as long as they are wide. Antennae are relatively short. 

*Cimex cavernicola* is reported from Russia and Turkmenistan [57]. It has slender hind femora (3.4 or more times as long as wide), long antennae, and a head width to third antennal segment ratio of less than 1.4 [17]. Little is known about this species and its worldwide dispersion.

*Cimex dissimilis* was first described by Horváth in 1910 [16]. *C. dissimilis* can be seen as a vagrant species that does not form a stable population with the local resident bat populations, but arrives occasionally and transferred by migrating bats [58]. *C. dissimilis* was recorded for the first time from Balkan Peninsula. It was reported frequently from 1968 to 1975 in the former Czechoslovakia [59]. Its current range of distribution has considerably extended to the south. In Italy, *C. dissimilis* was collected only on *Nyctalus noctula* [60]. It is proposed that *C. dissimilis* (Horvath, 1910), *C. stadleri* (Horvath, 1935), and *C. singeri* (China, 1938) are synonyms of *C. pipistrelli* (Povolný 1957). Nevertheless, *C. dissimilis* and *C. stadleri* are considered as valid species by other authors [40,54,61]. *C. dissimilus* (syn. *C. stadleri*) was collected in various European countries like Belgium, the former Czechoslovakia, Great Britain, France, Hungary, Netherlands, Switzeland, Poland, Russia, Byelorussia, and Ukraine [62]. *C. dissimilis* is morphologically characterized by bristles at middle of hemelytral pads with 0.1 mm length, which is longer than the distance between bristles [17].

*Cimex emarginatus* is present in the Balkan Peninsula, Bulgaria, Romania, and Greece (Simov et al., 2006). The taxonomic position of *C. emarginatus* remains unclear [17]. This species is similar to *C. adjunctus* and *C. brevis* in its chromosomal formula [17]. Regarding the shape and hairiness of the paragenital sinus, this species is similar to *C. lectularius* and *C. columbarius*. Moreover, *C. lectularius,* and *C. columbarius* differ from *C. emarginatus* by a fewer number of autosomes (13 bivalents autosomes) [63] and by a longer hind femora. *C. adjunctus* and *C. brevis* differ from *C. emarginatus* by the shape of the paragenital sinus (rounded with bristles), by a smaller Y chromosome, by longer bristles at the edges of the pronotum, and by a shorter hind femora (ratio of length/width of hind femur 2.1–2.8) [17]. The species of *Pipistrelli* group differ from *C. emarginatus* by the hairiness of the paragenital sinus (cleft and naked) and by the lower ratio of width to length of the pronotum (2.0–2.5) [58]. 

*Cimex flavifuscus* has been firstly reported in Eastern China [62]. It is identified by the serrate bristles on the outer sides and with scutellum having about 25 bristles on each side. It is considered to be a natural vector of *Trypanosoma scotophili* [64].

*Cimex himalayanus* is an ectoparasite of bats in the Himalayan region in India (Bhat, 1974). The description of this species was based on a single male specimen, collected while feeding on a *Myotls sillgorensls,* trapped in a mist net [37]. This species can be distinguished from other *Cimex* species by some morphological criteria, such as the presence of proportionate anger serrate setae on antennal segments, and on pronotal, hemelytral, and abdominal tergal margins, as well as the presence of minute setae on a discal area of pronotum [37].

*Cimex insuetus* has a geographical extension encompassing Thailand, India, and China [65,66]. It is similar to *Stricticimex* in particular with its developed pronotum, scattered long bristles, and paler color. However, experimental hybridization disclosed its relatedness with *C. hemipterus* [36]. It differs from *C. pulveratus* by its width to length ratio of the pronotum of less than 2, an elongated head and second antennal segment longer than the interocular space [35]. It has been reported as a vector of the Kaeng Khoi virus (KKV; family *Bunyaviridiae*, genus *Orthobunyavirus*), collected frequently in several caves in Thailand [66].

*Cimex japonicus* is present in Asia, particularly in Japan. It possesses scarcely serrate bristles and scutellum with about 12 bristles on each side [17].

*Cimex pipistrelli* is a typical ectoparasite of bats. It is morphologically differentiated among bat host species, but it does not reflect this genetically. Therefore, this proposes a possible morphological plasticity with high gene flow among populations associated with different host species [38]. Some cases of human biting, in the absence of its preferred host, are reported [17]. In 1957, Povolný proposed that *C. dissimilis*, *C. stadleri*, and *C. singeri* are synonyms of *C. pipistrelli*. Pringault [67] documented the presence of nonpathogenic trypanosomes flagellates in the gut of *C. pipistrelli* specimen collected in Europe. Later, Gardner et al. [68] reported the infection of this species by *Trypanosoma incertum* in the specimens, collected in Britain. *C. pipistrelli* possesses distinct characteristics that allow it to be identified morphologically. The longest bristles in this species are longer than the width of the first antennal segment, more than 0.13 mm. Moreover, bristles of abdominal tergites are mostly longer than the distance between bristles [17]. 

*Cimex pulveratus* has been described based on two bug specimens (a male and a female) collected on *Hypsugo pulveratus* (Chinese pipistrelle) in Vietnam [69]. It is characterized by a small size (3.8 mm), short pubescence, pronotum width to length ratio above 2.5, and rounded hind margins of the hemelytral pads on inner halves and anterior and posterior bristles more than 100 μm [69]. It is similar to *C. lectularius* and *C. hemipterus* based on its coxal spur, width to length ratio of pronotum more than 2.5, as well as its head shape (wider than long), respectively [69].

*Cimex singeri*, described in the western Palaearctic region, has an unclear taxonomic status. Povolny considered it as a synonym of *C. pipistrelli* [45,62].

*Cimex stadleri* was first described in Germany by Horvath in 1935. Pericart [44] and Wendt [22] proposed the synonymy between “dali *C. stadleri*” (Horvath 1935) and *C. dissimilis* (Horvath 1910) [42,43], while others accepted it as a monotypic species of *C. pipistrelli* [22,24,43]. In this species, the bristles present at middle of the hemelytral pads are less than 0.1 mm and shorter than the distance between bristles (Usinger, 1966) [17]. 

*Cimex usingeri* is a species infesting habitat of *Rhinolophus rouxi* (*Chiroptera*, Rhinolophidae) in India [36]. This species resembles *C. burmanus* in having a short third antenna segment, pronotum with long nonserrate marginal bristles and stout hind femora. Nevertheless, it differs from *C. burmanus* by longer bristles on pronotal margin, longer bind femora, and paragenital sinus, surrounded by a small bare area [36]. 

## 4. Fossil Evidence and Evolutionary History of Bed Bugs

The oldest fossil evidence of Hemiptera (true bugs) belongs to the *Aviorrhyncha magnifica* (Aviorrhynchidae, Euhemiptera) (dated to ~310 MYA) which, together with bed bugs, (Cimicidae family) constitute the infraorder Cimicomorpha [70]. Fossil evidence of *Quasicimex eilapinastes*, corresponding to an ancestor of the Cimicidae family, was collected in Burma amber and dates back to 100 MYA (million years ago) [71]. Bats have long been considered as the zoophilic ancestor host of bed bugs and the oldest bat fossil dates back over 30 MYA (Early Eocene) [72]. A recent fossil-based phylogenetical investigation firmly demonstrates that the bugs have evolved long time before bats and have parasitized them on several occasions. This phylogenic analysis placed ancestral cimicidae to 115 MYA, therefore 30 to 50 MYA prior to the bats’ emergence [73,74]. The identity of the ancestral host(s) from which bats were colonized repeatedly is unknown [74]. Then, lineages were later frequently populated bat and bird lineages [73,74,75,76]. This hypothesis is in accordance with previous theory which reports the hematophagous species of Polyctenidae as the sister group of Cimicidae [11,77]. Interestingly, the clades that encompass two current major prevalent ectoparasites, *C. lectularius* and *C. hemipterus*, separated 47 MYA. Consequently, this hypothesis is against the notion implying the evolutionary trajectories of *Homo* caused their divergence, knowing that the split between *H. sapiens* and *H. erectus* clades dates from 1.6 MYA [74]. 

**Figure 4:**
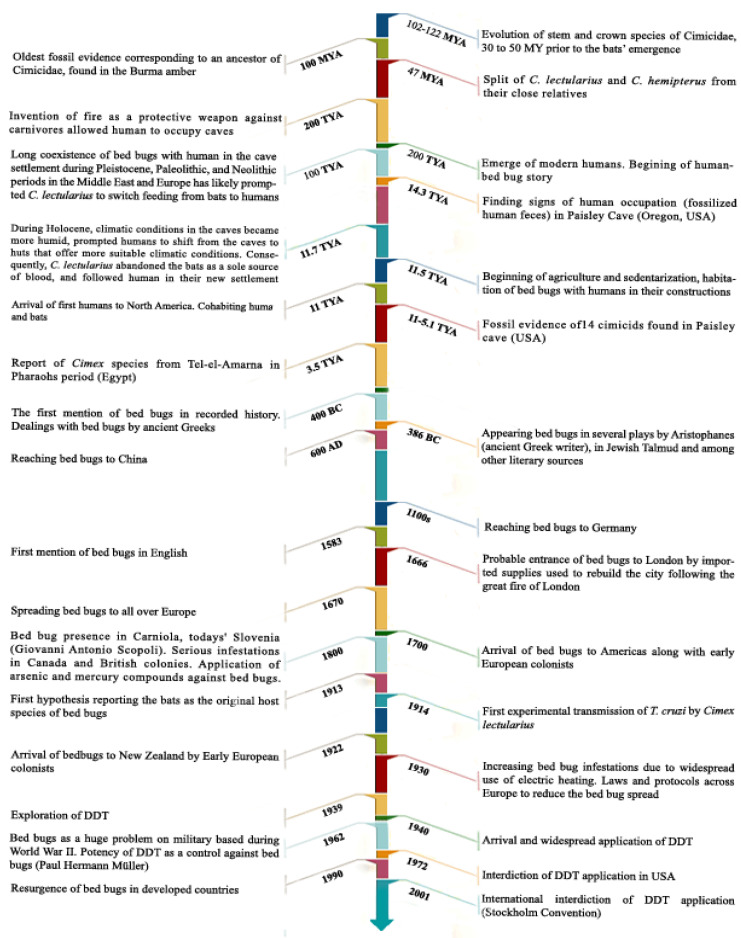
History of the description of bed bugs according to literature [17,65,74]. MYA: Million Years Ago. TYA: Thousand Years Ago.

Other fossil evidence, attesting the presence of bugs was collected during archaeological investigations in the Paisley cave in south-central Oregon, USA [49]. In this cave, 14 cimicids (Hemiptera: Cimicidae) were collected. The age of these bug specimens varied from 11,000 to 5100 years ago (Figure 4). Nine of these specimens belonged to the three Nearctic Cimicidae species, which include *C. antennatus* (Usinger and Ueshima 1965), *C. latipennis* (Usinger and Ueshima 1965), and *C. pilosellus* (Horvath 1910), and the remaining five individuals were too degraded to make an accurate identification [49]. A detailed history of bed bugs descriptions is given in Figure 4.

## 5. Historical Human–Bed Bug Cohabitation

Cimicid bugs typically remain in the hosts’ roosts to get blood meals [17,78] and would have a low inherent capacity for dispersal to contiguous structures, being rather dependent on their hosts for dispersal [17]. Clades of *C. lectularius* parasitizing humans had diverged at least 5–10 MYA before the oldest known *Homo* species, the spatial and temporal coexistence of several lineages of hominids allow several host shift scenarios. Therefore, no matter when hominids first entered caves, bat, and bird parasitizing *C. lectularius* were already present to exploit this new opportunity [74]. The opportunity for *C. lectularius* to increase host preference dates from the late Pleistocene when humans, bats, and bed bugs inhabited together in the caves of the Northern Mediterranean and Central Asia [58,79]. Later, during the Holocene, the more humid climatic conditions in the caves may have prompted humans to shift from caves to huts that offer more suitable conditions [58,80]. Consequently, *C. lectularius* followed human in their new settlement [54,81,82]. Bugs were found in human dwellings in Turkmenia, where bats and bugs cohabit in caves [83,84]. The invention of fire as a protective weapon against carnivores allowed humans to occupy caves or rock shelters before approximately 250 TYA in Europe [85]. About 100 TYA in the Middle East, humans cohabited with bats (*i.g., Myotis and Rhinolophus*) in caves and lived under similar conditions through the last Ice Age (12,000 B.C.) as well [86]. Later, approximately 11,500 years ago, at the beginning of the agriculture and sedentarization, *C. lectularius* continued to cohabitate with humans in their habitations [87]. The relationship between humans and bed bugs became more established, when humans transitioned from their transient lifestyle as hunter-gatherers to a more stable community of farmers, living in villages 8000–5000 BC [54,88]. This period corresponds to an expension in trade and travel along with a migration of the human populations from small villages to cities. Some bat species, like *Eptesicus fuscus* and *Myotis lucifugus*, might have roosted in human-dwelling buildings [89,90], allowing bat parasitings bugs to cohabite with humans [17,54]. Roost-switching of bats, caused by high bat bug load, is also a possible cause for the association of bats with *C. pipistrelli* [91]. On the other side, it is hypothesized that both *C. lectularius* and *C. hemipterus* originate from the old world regions [17,61]. This hypothesis was proposed based on morphological (narrowly clefted paragenital sinus in old world bed bugs compared to rounded notch in new world ones) and linguistic (no word for bed bug in any native American language) evidences, together with the known geographical distribution of *Cimex* species [17]. Afterwards, bed bugs probably spread from the Middle East and North Africa and then through Europe and Asia. A probable scenario explaining the passage across the Mediterranean Sea would be an infestation of the trading ships during the Bronze Age. The first official evidence of the close relationship between bed bugs and humans date back to 3550 which was discovered in the archaeological digs in the south of Cairo [87]. The earliest records of bugs associated with human settings in Greece dates from 400 BC [54,92]. The intense urban and economic developments during the Roman period have probably allowed *C. lectularius* to be transported by ships. Then, the increasing urbanization and the improvements in housing that have taken place during the post-medieval period have offered the suitable condition for *C. lectularius* to further expend its distribution range [87,93]. In England, the first evidence of bed bugs presence was discovered in a Roman pit that dated from the second century AD [87]. Bed bug infestations were reported in England in 1583, and became common during the 17th and 18th centuries. In Iraq, bugs were known in the ninth centuries, according to the description of the Arabic scholar al-Jahiz, who wrote: ‘They feed on warm blood and have a crazy preference for man’. Other records disclosed the presence of bed bugs in Italy, 77 AD, in China, 600 AD, in Germany during the 11th century, France during the 13th century, and England, by the late 1500s [54,92]. The rise in cases of infestation, in northern European cities in the early 20th century, is likely to be due to the increase use of central heating [94]. In North America, the first description of bed bugs dates from the 1600s [51]. These events highlight the expansion of bed bugs, which largely correlates with the growth of civilization and have offered the opportunity to this ectoparasite to proliferate and disseminate all over the world.

Considerable genetical and morphological divergence have been recorded between *C. lectularius* populations associated with humans, and those collected from synanthropic bats. Interestingly, *C. lectularius* collected from humans and bats show striking differences in their feeding frequency and mortality when they are allowed to feed on their irrespective host (e.g., bats for *C. lectularius* collected on human and human for *C. lectularius* collected from bats) [95]. A highly limited gene flow between these two populations is disclosed, with a prevailing gene flow direction from human-associated *C. lectularius* populations to that of bat-associated ones [96]. Altogether, morphological, molecular, and physiological evidences depict independent evolutionary histories of *C. lectularius* populations, feeding on bats to those feeding on humans [95,96]. Indeed, molecular evidence supports the divergence between these two lineages approximately 245 TYA, as early as the modern human dispersion out of Africa [97]. 

## 6. Current Worldwide Distribution of Bed Bug Species with Emphasis on the Cosmopolitan Species

Bed bugs, mostly *C. lectularius*, have undergone a recent and rapid global resurgence. During the last two decades, bed bug infestations have been frequently reported to affect private houses, hotels, apartments, and universities, as well as public transportation (e.g., planes, trains, and buses) [98].

### 6.1. Middle East

**Figure 5:**
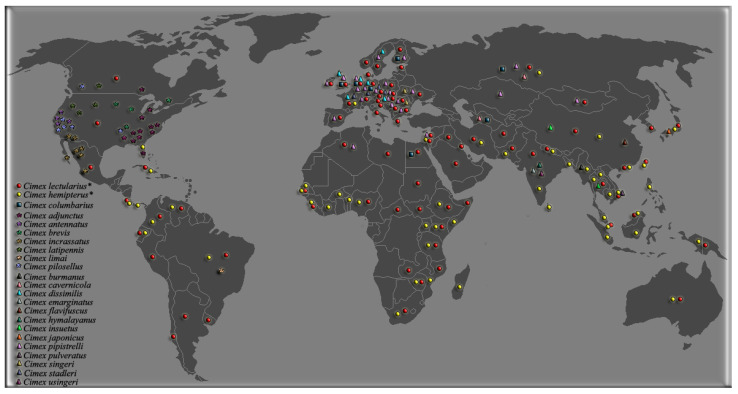
Global dispersion of 23 species belonging to the *Cimex* genus. The medically important species are indicated by an asterix. The countries with no pinpoint means no data is available. The pinpoint location represents only the presence of *Cimex* species with no indicative geographical position of the infestations.

It is believed that bed bugs might have originated from the Middle East and areas along the Mediterranean Sea [54]. An early investigation performed by Patton [99,100] proposed that in Mesopotamia, *C. hemipterus* was the sole species inhabiting Indian communities in cities like Basra and Baghdad. More recently, Abul-hab [101] reported the presence of *C. lectularius* in Iraq. In Israel, *C. hemipterus* together with *C. lectularius* represent a significant health problem for the population. Rosen et al. [102] reported the occurrence of *C. hemipterus* in the poultry farms of this country. *C. lectularius* is the predominant species encountered in this region and *C. hemipterus* being reported in sympatry in some restricted locations [65,103] (Figure 5). 

### 6.2. Asia

In Asia, a resurgence of bed bug infestations has occurred along with their resurgence in Europe, America, and Australia. In East Asia, the presence of bed bugs is attested in China at least 600 BC [92]. Based on Chinese and Japanese medical texts, at least in the 18th century, bed bugs were much more prevalent in China than in Europe and were probably rare, if not absent, in Japan [65]. *C. hemipterus* and *C. lectularius* are described in Asia and are in sympatry in some Southeastern Asian countries, i.e., Japan, China, and India [104,105]. In Japan, the first report on the presence of bed bugs dates from the 1860s (at the end of the Edo period) in the Southern part of the country, where international trade took place. The availability of cheap accommodation for the homeless and tourists was another probable reason for the rapid spread of bed bugs in this country (Motokazu Hirao, Japan Pest Control Association, unpublished results). In China, there is no report on bed bugs’ occurrences before 1949, mainly because of local conflicts. In 1953, the co-occurence of *C. lectularius* and *C. hemipterus* was noticed [106]. *C. lectularius* is the most prevalent species in China, whereas *C. hemipterus* is reported only in the southern part of the country (i.e., Guangdong province and Guangxi Zhuang Autonomous Region) [104]. In Southeastern Asia, *C. hemipterus* appears to be the primary species involved in infestations. In Malaysia, several factors may have played an important role in the recent resurgence of bed bugs, including the increase number of legal and illegal migrant workers [107]. In India, *C. lectularius* (in the north of India) and *C. hemipterus* (in the south of India) are commonly reported. They are considered as responsible for nuisance, among Indian armed forces and a declining cause of general health [108]. In addition, other eight bat parasitic species are reported in India, including *C. himalayanus*, *C. insuetus*, *C. usingeri*, and *C. pipistrelli* [65,109].

### 6.3. Europe

There is a long history of bed bug presence in Europe, with one of the first primitive descriptions of bug infestation in the early Greek literature [110]. It is thought that bed bug infestations in Europe have originated from the Middle East [17]. It seems most plausible that *C. lectularius* arrived in Europe via the belongings of early humans [65]. Fossil records of bed bugs in Europe date back to the Roman (1st century) and the medieval period (10th century) [111]. In England, the first records of infestation caused by *C. lectularius* dates from 1503 and the official report of bed bug infestation in the UK was in 1583 [17]. In the 1700s, reports showed that they were abundant in the coastal cities and had a lower occurrence in central regions. Its extension to the Nordic countries would have been done later, in the 19th century, via old sailing ships supposed to be heavily infested of bed bugs e.g., in Sweden [112]. In Europe, the spread of *C. lectularius* is attributed to the massive migrations of people towards urban areas during World War I (WWI) [17]. Beside *C. lectularius*, Horváth has described a new species namely *C. dissimilis* in Hungary and provided an identification key to the known Palaearctic species of cimicids [16]. Some years later, *C. stadleri* was described in the former Czechoslovakia [40]. In 1939, about four million people were subjected to bed bug infestations in London [17]. Since 1939 onwards, due to the introduction of residual insecticides, bed bug infestations declined. In 1948, an extensive survey of public buildings in Berlin (Germany) showed that bed bug infestation rates were as high as 40% in the city centers, decreasing to around 2% in the suburbs [113]. In Denmark, since 1945, it is mandatory to perform a survey for bed bugs when people leave their houses [114]. For most central Europe countries, very little is known about the bed bug occurrence after WWII, suggesting the absence of bed bugs infestation due to *C. lectularius* in these regions (Václav Rupeš, former head of the Department of Desinfection in Prague, unpublished document). In the USSR, bed bug infestation rates declined much later than in other European countries. After WWII, due to huge destruction, a rise in bug infestation was recorded [115]. Because of the high rate of bed bug infestation in 1958, the USSR government engaged actions to control it. Thanks to this strategy, the infestation rate decreased to 35% in 1960 [115]. After WWII it is thought that the use of the insecticide DDT led to a sudden and sharp decline in infestation cases worldwide, although this was later questioned by Reinhard [116]. Indeed, from the late 1960s to the early 1970s, the number of bed bug treatments remained approximately unchanged [117]. In the 1990s, most of European countries have experienced a resurgence of bed bugs infestation (primarily by *C. lectularius*). A report on the resurgence of bedbug infestation in England has been published by Birchard in 1998, but the evidence on their worldwide resurgence was provided by Boase [118]. In 1996, Péricart mentioned the occurrence of *C. pipistrelli* in European countries, such as England, Ireland, and Germany, and probably in Sweden, and proposed that only *C. pipistrelli* and *C. dissimilis* (= *C. stadleri*) are present in Western Europe. Melber [119] reported the presence of *C. dissimilis* and *C. pipistrelli* in Germany. In central Europe, bug species from the *Pipistrelli* group are associated with bats or birds [120]. In the 2000s, Denmark and Sweden reported a significant increase of bed bug infestations [121]. In Sweden between 2000 and 2006 a 100% increase in infestation was recorded by pest control operators [121]. In Denmark, a gradual annual increase in bed bug infestation was observed during the same period [121]. The resurgence of *C. lectularius* across western and central Europe appears actually to be stable with a relatively slow rate of increase that is in contrast to the sharp increase, reported in Nordic countries. Considering that Paris together with other main cities in Western Europe like London and Berlin are as the first destinations for tourism, it is possible that *C. hemipterus* has been probably imported by the passengers from the countries infested by this species. Recently, cases of *C. hemipterus* infestation were reported from France [122] and Italy [123]. The *Cimex* species currently reported in Europe are: *C. lectularius*, *C. hemipterus*, *C. pipistrelli*, *C. columbarius, C. dissimilis*, and *C. emarginatus* (Figure 5).

### 6.4. Africa

One of the most ancient evidence on the presence of *C. lectularius* in Africa comes from the Tell el-Amarna (Egypt) region and dates from the Pharaohs period (1323–1350 BC) [87]. During WWI, bed bug infestations of the soldiers’ helmets were reported during the East African military campaigns [92]. After WWII, cases of infestation declined in a number of African countries; but in some countries, high numbers of *C. hemipterus* and *C. lectularius* infestations were noticed during the 1970s and 1980s [124]. One of the first reports describing insecticide resistance of *C. hemipterus* against Dieldrin (an organochloral insecticide) was noticed in Zanzibar Island [125]. Afterward, several cases of resistance were reported in African countries, i.e., Kenya, Somalia, Egypt, Gambia, Zimbabwe, Congo, and South Africa [126,127]. Such a sharp increase in insecticide resistance rate would imply either a vast and rapid dispersion of bed bugs in African countries or a multiple emergence of resistant bugs. In “La Reunion” Island, according to the data collected by pest control intervention programs, infestations by *C. hemipterus* are increasing [7]. At present, the lack of an exhaustive entomological and epidemiological surveillance in Africa led to an underestimation of the real total number of infestations [124,128]. In the continent of Africa, three species (*C. lectularius*, *C. hemipterus*, and *C. pipistrelli*) are present.

### 6.5. North and South America

Canada and USA have a long history of bed bug infestations. Due to the temperate climate of these countries, most infestation cases are restricted to *C. lectularius*. Nevertheless, until now, 16 species, including *C. antennatus*, *C. adjunctus*, *C. brevis*, *C. incrassatus*, *C. latipennis*, and *C. pilosellus* are reported to be present in the United States and Canada [17,129]. Nevertheless, *C*. *lectularius* remains the major ectoparasite bug species found in North America [65]. The origin of infestation can be dated back to the early European colonists in the 17th century. By the 1900s, bed bugs were extremely common before WWII in the USA. They are mainly observed in buildings with poor hygiene and inhabited by transitory residents [130]. In the 1940s, the application of DDT led to a near elimination of bugs’ infestation in North America. However, in recent decades, infestation cases have increased, likely due to international transportation and diffusion of insecticide resistance [131]. The first case of control failure of DDT against *C. lectularius* was reported in 1947 from the barracks of the Naval Receiving Station in Hawaii [132]. In Canada in 1748, presence of bed bugs was thought to be due to the trade and travel of indigenous people with English colonists. Seven species are recorded in Canada and four are bat parasitic species [51]. They include *C. latipennis*, *C. brevis*, *C. adjunctus*, and *C. pilosellus* [51,133,134]. 

Latin American countries, i.e., Argentina, Chile, Colombia, Ecuador, Panama, Peru, and Venezuela, have encountered bed bug infestations to a lesser extent. Nevertheless, in all these countries, bed bug infestations are not mostly reported, because they are considered as a nuisance with relatively minor health issues [65]. *C. lectularius* and *C. hemipterus* are of the species present in South America [65,135]. Lutz and Machado, more than a century ago, published the first report of a *C. lectularius* infestation in Brazil [136]. At that time, *C. lectularius* and *C. hemipterus* were commonly found in Brazil, with *C. hemipterus* being much more frequent than *C. lectularius* in overcrowded centers and rural regions [137]. *C. lectularius* is predominantly distributed in southern region and associated with the intensive European immigration. In Argentina, four species are reported: *C. lectularius*, *Propicimex tucmatiani*, *Latrocimex spectans*, and *Bucimex chilensis* [138,139]. In various cities of Argentina, Chile, and Uruguay, a survey performed via a questionary revealed an occurrence of 84% [140]. The majority of the reports were collected from insect control companies by local authorities and hotels [92]. In these countries, bed bug infestations that were often reported in the 1950s have declined due to control strategies implemented against mosquitoes. Nevertheless, they re-emerged in the 1990s, simultaneously with the re-emergences reported in Europe and North America. Due to the co-occurrence of bed bug species together with triatomine bugs (vector of the Chagas disease) in this region, bug infestations have been and remain a center of attention.

### 6.6. Australia

It is thought that the origin of bed bug infestations in Australia correspond to imported cases by early colonialists via their sailing vessels during the 18th century [141]. This hypothesis is attested by the writings of an early Australian naval explorer, Matthew Flinders, who reported infestations of his ship in 1803 [142]. Then, bed bugs disseminated quickly and were a common problem in the 1920s. In the late 1930s and early 1940s, bed bug infestations were highly prevalent even in health centers and hospitals. At the beginning of WWII due to the introduction and the widespread use of organochlorine insecticides, the infestations were just reported sporadically [143]. After the 2000s, in particular during 2001–2004, a sharp increase of 700% rise in bed bug infestation treatment was reported [144]. A rise in alerts of bed bugs’ presence in luggage was acertained by the national quarantine inspectors from 1986 to 2003 [144]. A high infestation rate (74%) is noticed from 1999 to 2003 by these inspectors. *C. lectularius* (mainly in southern part of Australia) and *C. hemipterus* (mostly in central and north of the coutry) are present in this region and are sympatric in some locations [6,101].

An illustrative map highlighting the worldwide distribution of *Cimex* species is given in Figure 5. Detailed information of the worldwide dispersion of bed bugs together with quoted references in each country is given in Table S2. In the map, the absence of pinpoint indicates that no data are currently available for the country. As illustrated in the map, bed bugs are known to be present in more than half of the countries.

## 7. Conclusions

*Cimex* species have emerged 115 MYA with an “unknown ancestral host”, over 30 and 40 MY before bat and *Homo* emergences, respectively. Bed bugs and humans have a long history of coevolution and according to Usinger [17], contact between *C. lectularius* and humans may have originated in the Middle East in the caves that were inhabited by hunter-gatherers and bats. Evidences attesting the presence of *Cimex* species in Paisley cave in the USA that date back to 11,000 years ago [68], therefore prior to the agricultural period and sedentarization, suggests that similar contacts between human and cimicid bugs might have likely occurred in North America. The widespread international transport and trade of secondhand materials, military conflicts, and immigration have favored the diffusion of several *Cimex* species, particularly in Europe and North America. Up until the present, 23 species belonging to the *Cimex* genus have been described although the taxonomic position of some of these taxa is still debatable. Among the mentioned species, *C. lectularius* is the most prevalent species worldwide. Nevertheless, little is known about the origin and evolution of *Cimex* species despite their incredible adaptive and disseminates capacity.

## Supplementary Materials

The following are available online at https://www.mdpi.com/1660-4601/17/12/4576/s1, Table S1: Prisma checklist of article database investigated in the present study, Table S2: Worldwide dispersion of different *Cimex* species, present in different countries with their quoted references.
